# HOXB13 mutations and prostate cancer in Poland

**DOI:** 10.1186/1897-4287-10-S4-A30

**Published:** 2012-12-10

**Authors:** Wojciech Kluźniak, Dominika Wokołorczyk, Jan Lubiński, Cezary Cybulski

**Affiliations:** 1 Department of Genetics and Pathomorphology of the Pomeranian Medical University, Szczecin, Poland

## 

Recently, HOXB13 has been established as a prostate cancer susceptibility gene in North America, with a relative risk associated with a single missense mutation of about 20. Ewing et al., sequenced over 200 genes in a prostate cancer linkage region at 17q21-22 among 94 probands of prostate cancer families, and found a recurrent mutation in the HOXB13 gene (G84E) in four families. The mutation co-segregated with prostate cancer. The geographical and ethnic extent of this founder allele has not yet been determined. We assayed for the presence of the G84E mutation in 3515 prostate cancer patients and 2604 controls from Poland. The G84E mutation predisposes to prostate cancer in Poland. We expect that the G84E founder mutation might be present in other Slavic populations.

